# Efficacy of desferrioxamine mesylate in intracerebral hematoma: a systemic review and meta-analysis

**DOI:** 10.1007/s10072-022-06324-0

**Published:** 2022-08-25

**Authors:** Kai Zhao, Jing Li, Qiang Zhang, Mingfei Yang

**Affiliations:** 1 Graduate School, Qinghai University, Xining, 810016 Qinghai People’s Republic of China; 2 Department of Community Health Education, Institute for Health Education of Qinghai Province, Xining, Qinghai 810000 People’s Republic of China; 3 Department of Neurosurgery, Qinghai Provincial People’s Hospital, No. 2 Gonghe Road, Xining, 810007 Qinghai People’s Republic of China

**Keywords:** Perihematomal edema, Intracerebral hematoma, Desferrioxamine mesylate, Meta-analysis, Treatment

## Abstract

**Background:**

Previous meta-analysis had concluded that desferrioxamine mesylate (DFO) could effectively treat intracerebral hematoma (ICH) in animal models. We hope to confirm that DFO could treat ICH patients effectively through the systemic review and meta-analysis of clinical researches.

**Method:**

Data extraction included hematoma volume (HV), reduction of National Institute of Health Stroke Scale (NIHSS) scores, and relative perihematomal edema (RPHE). The standard mean difference (SMD) and 95% confidence interval (95%*CI*) were calculated by fixed effects model. I-square (*I*^2^) statistic was used to test the heterogeneity. All *p* values were two-side with a significant level at 0.05.

**Results:**

Five randomized controlled trials were included in the meta-analysis, which included 239 patients. At 7 days after onset, there was significant difference of RPHE development (− 1.87 (− 2.22, − 1.51) (*I*^2^ = 0, *p* = 0.639)) and significant difference of HV absorption (− 0.71 (− 1.06, 0.36) (*I*^2^ = 17.5%, *p* = 0.271)) between DFO and control groups. There was significant difference of reduction of NHISS scores (0.25 (0.05, 0.46) (*I*^2^ = 0, *p* = 0.992)) between DFO and control groups at 30 days after onset.

**Conclusion:**

DFO reduced HV and perihematomal edema in ICH patients at 7 days after onset and improve neurological function at 30 days after onset efficiently and safely. DFO might be a new route of improving treatment of ICH.

## Introduction

Intracerebral hematoma (ICH) resulted from craniocerebral injury or hemorrhagic stroke is a common disease in nervous system. Patients have poor prognosis due to the compression of the brain lobe by hematoma lesion and toxic effects of hematoma [1]. Besides, perihematomal edema (PHE), the edema around hematoma, can aggravate condition of ICH patients at early stage [2]. Besides, along with the hematoma absorption, ferric ion can result in cortical iron deposition, which can lead to permanent nerve damage [3]. In-time reduction of PHE and hematoma volume (HV, unit: ml) could effectively reduce the mortality and disability rate of patients.

Desferrioxamine mesylate (DFO) is a kind of chelator that is clinically used to treat iron poisoning and iron overload [4]. Chelates consist of DFO and ferric ion, which could be excreted completely through urine and feces [5]. Therefore, iron deposited in organs could be reduced. Iron in plasma or cell, such as ferric ion in ferritin and hemosiderin, can be chelated, except iron in transferrin and hemoglobin.

Thus, DFO might be useful in treatment of ICH because it could accelerate hematoma absorption. The meta-analysis of animal experiments has concluded that DFO could effectively treat ICH in animal models [6]. However, the efficacy of DFO in ICH patients has not been assessed systemically. In this study, we conducted a meta-analysis of recent randomized controlled trials to confirm our hypothesis that DFO could effectively promote reduction of HV and PHE and improve neurological function in ICH patients.

## Methods

### Literature search

Three open electronic databases of PubMed, EMBASE, and Cochrane were searched. The strategy of literature search was: ((“Deferoxamine” [Mesh])OR ((((((((((((((((Desferal [Title/Abstract])OR(“Methanesulfonate, Deferoxamine” [Title/Abstract]))OR(“Deferoxamine Methanesulfonate” [Title/Abstract]))OR(“Mesylate, Deferoxamine” [Title/Abstract])) OR(“Deferoxamine Mesylate” [Title/Abstract]))OR(“Mesilate, Deferoxamine” [Title/Abstract]))OR(“Deferoxamine Mesilate” [Title/Abstract]))OR (“Mesylate, Desferrioxamine B” [Title/Abstract]))OR(“Desferrioxamine B Mesylate” [Title/Abstract]))OR(“Desferroxamine” [Title/Abstract]))OR(“Deferrioxamine B” [Title/Abstract]))OR(Deferoximine [Title/Abstract]))OR(“Desferrioxamine B” [Title/Abstract]))OR(“Deferoxamine B” [Title/Abstract]))OR (Desferioximine [Title/Abstract]))OR(Desferrioxamine [Title/Abstract])))AND((“Cerebral Hemorrhage” [Mesh])OR(((((((((((((((“Hemorrhage*, Cerebral Brain” [Title/Abstract])OR(“Cerebral Brain Hemorrhage*” [Title/Abstract]))OR(“Brain Hemorrhage*, Cerebral” [Title/Abstract]))OR(“Hemorrhage*, Cerebral” [Title/Abstract]))OR(“Cerebral Hemorrhages” [Title/Abstract]))OR(“ICHs” [Title/Abstract]))OR(“Hemorrhage*, Intracerebral” [Title/Abstract])) OR(“Hemorrhage, Intracerebral” [Title/Abstract]))OR(“ICH” [Title/Abstract]))OR(“Parenchymal Hemorrhages, Cerebral” [Title/Abstract]))OR(“Parenchymal Hemorrhage, Cerebral” [Title/Abstract])) OR(“Hemorrhage*, Cerebral Parenchymal” [Title/Abstract]))OR(“Cerebral Parenchymal Hemorrhage*” [Title/Abstract]))OR(“Hemorrhage*, Cerebrum” [Title/Abstract]))OR(“Cerebrum Hemorrhage*” [Title/Abstract]))).

### Inclusion and exclusion criteria

The inclusion criteria consisted of the following: (1) Language and regions of articles were not restricted; (2) date of publication was up to December 31, 2021; (3) randomized controlled trials; (4) patients suffered from ICH, which was brain space occupying lesion caused by hematoma including craniocerebral injury or hemorrhagic stroke; (5) DFO was used as the clinical intervention; and (6) outcomes involved changes of neurological function, HV, and PHE. The exclusion criteria consisted of the following: (1) duplication; (2) reviews, comments, letters, case reports, protocols of clinic trials, and conference papers; (3) animal experiments; and (4) articles with none-related topics.

### Quality assessment and data extraction

The quality assessment of included articles was performed via the Cochrane Collaboration’s Tool of Assessing Risk of Bias by the Review Manager version 5.3 software. When 2 articles were assessed to the same scores, we considered the one with the more number of participants had the higher quality.

The outcome measurement of this meta-analysis was the effect of DFO on ICH patients. Changes of HV and absolute volume of PHE (PHEV) at 7 days after onset were extracted, which could show the effect of DFO in ICH patients directly. But PHEV could not reflect the relationship that PHE was resulted from hematoma. Relative PHE (RPHE) could remedy the deficiencies, which was calculated by the formula of (PHE volume)/(the total volume of hematoma and PHE) [7]. We preliminarily chose the National Institute of Health Stroke Scale (NIHSS) scores at 30 days after onset to measure recovery of nerve function. In addition to the three continuous variables above (HV and RPHE at 7 days after onset, and NIHSS scores at 30 days after onset), some confounders, which might result in errors, were also extracted, including causes of ICH, characteristics of subjects, period of treatment, and conclusions of studies.

### Statistical analysis

Mean and standard deviation (SD) were used to perform statistical description of continuous variables of normal distribution. Statistical difference of data before meta-analysis was tested by One Way Analysis of Variance (ANOVA) using SigmaStat version 4.0 software. If there was no statistical difference, data was directly used for meta-analysis. The differences of data at baseline and other time points were manually calculated. Meta-analysis was performed using Statistics and Data Science version 15.1 software. The overall standard mean difference (SMD) with its 95% confidence interval (95%*CI*) was calculated by fixed effects model. I-square (*I*^2^) was used to test the heterogeneity. Sensitivity analysis, which was the way to checkout the stability of overall results, consisted of two methods that overall SMD of rest studies were performed after the study with the highest quality omitted or the fixed effects model was switched to random effects model. Funnel plot and Egger’s regression were used to analyze publication bias. The method to attenuate heterogeneity and enhance sensitivity was the deletion of studies with publication bias or the lowest quality and subgroup analysis. All *p* values were two-side with a significant level at 0.05.

## Results

### Study selection and characteristics

**Fig. 1:**
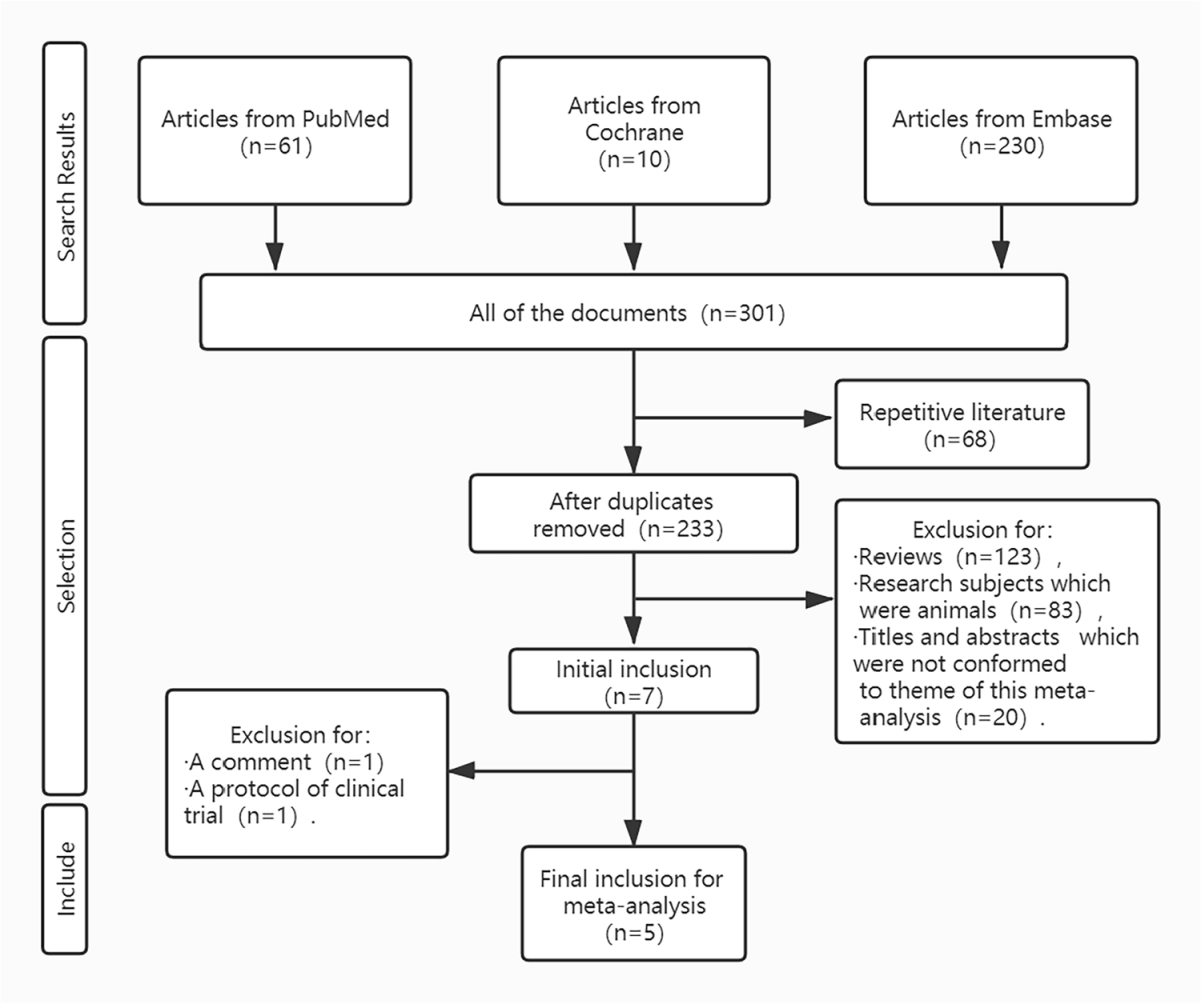
Flowchart of article screening

**Fig. 2:**
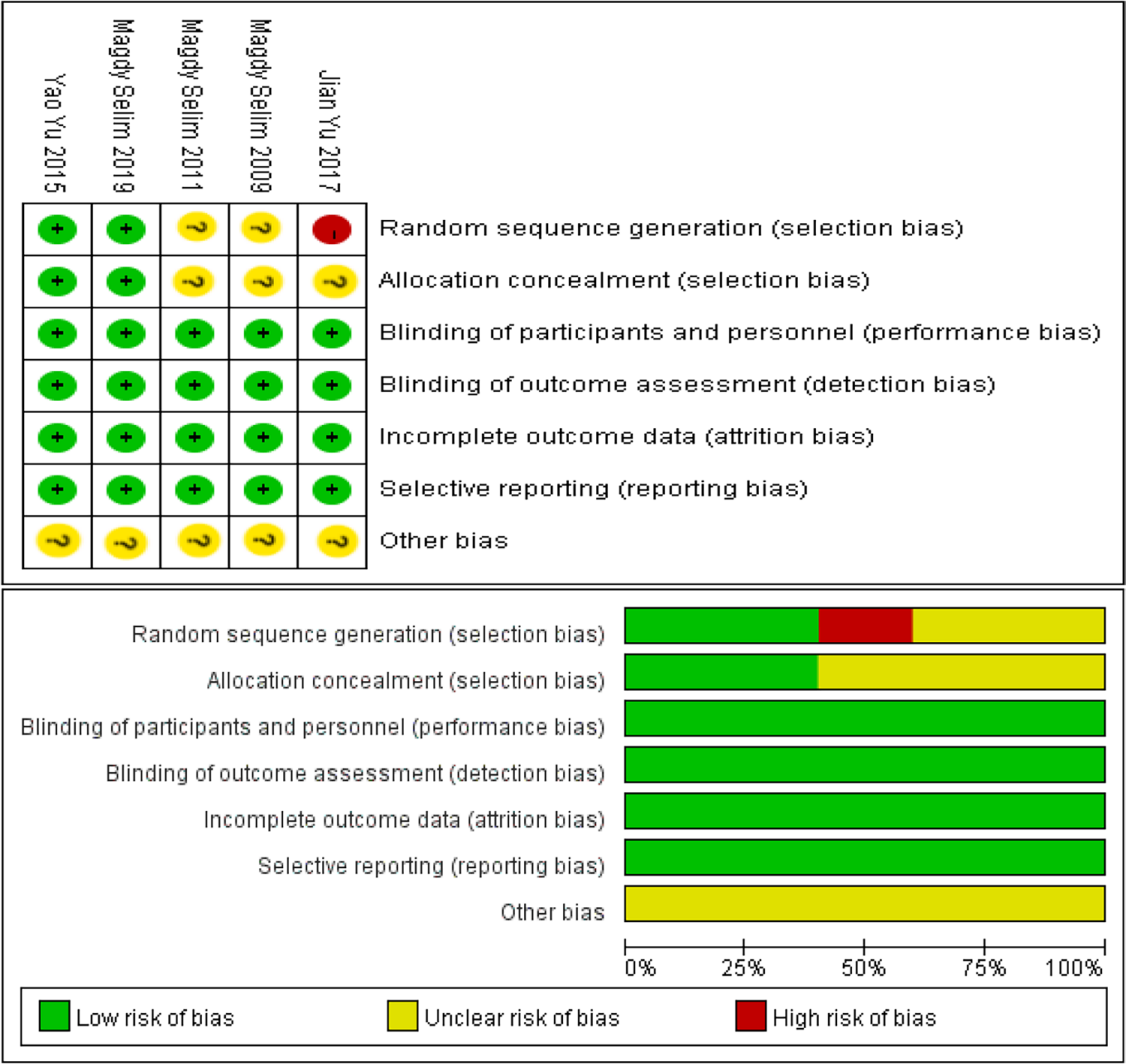
Assessment of article quality

Totally, 301 articles were retrieved from three databases according to the search strategy. After screening articles according to the inclusion and exclusion criteria, five articles of randomized controlled trials were included ultimately (Fig. 1). Some clinical trials have not been completed [8 9]. Figure 2 showed the assessment of quality of articles. In random sequence generation, one study [10] was of high-risk bias and two studies [11], (Selim M et al. 2009) were of unclear risk. Three studies [10, 11, 12] were evaluated to have unclear risk bias in allocation concealment. There was no bias in performance, detection, attrition, and reporting. All of studies had unclear risk bias in other biases.

Articles included were published between 2009 and 2019 (Table 1). Subjects of the experiment group (*n* = 239) were aged 39 to 81 years, including 47 cases of craniocerebral injury and 192 cases of hemorrhagic stroke. Time of treatment was 3 consecutive days after hospitalization except for one study of 5 days [10]. The dose of DFO was 20 mg/kg/d in one study [10], 32 mg/kg/d in two studies [13, 14] and 6–72 mg/kg/d in one study [11]. Maximum daily dose was 6000 mg in three studies [11, 13, 14] and 2000 mg in one study [10]. Especially, one study had the dose of 500 mg/d without the reference of patients’ weights [12]. Regarding to routes of administration, one study selected intramuscular injection [12]; one study selected intravenous injection [13]; and other studies selected intravenous infusion. According to conclusions of articles, two studies suggested that DFO might not improve the prognosis or hematoma absorption [13, 14]; however, three studies indicated that DFO might have potential neuroprotective effects via acceleration of hematoma absorption and inhibition of PHE [10, 11, 12].

**Table 1:** Characteristics of included studies

The first author and publication year	Participants	Causes of hemorrhage	Age (mean ± SD)	Sex (male%)	Drug dose	Route of administration	Time of treatment	Conclusions
Selim M 2010 [12]	7	Hemorrhagic stroke	75.14 ± 6.36	75.0%	500 mg/d	Intramuscular injection	3 consecutive days after hospitalization	DFO could exert potential neuroprotective effects in stroke patients
Selim M et al., 2011 [11]	20	Hemorrhagic stroke	69.00 ± 10.00	60.0%	7–62 mg/kg/d, maximum daily dose: 6000 mg/d	Intravenous infusion	3 consecutive days after hospitalization	Consecutive daily infusions of DFO after ICH were feasible, safe, and well-tolerated
Yu Y et al., 2015 [13]	21	Hemorrhagic stroke	64.2 ± 9.50	N/A	32 mg/kg/d, maximum daily dose: 6000 mg/d	Intravenous injection	3 consecutive days after hospitalization	Deferoxamine mesylate might slow hematoma absorption and inhibit edema after ICH
Yu J et al., 2017 [10]	47	Craniocerebral injury	53.36 ± 14.07	80.9%	20 mg/kg/d, maximum daily dose: 2000 mg/d	Intravenous infusion	5 consecutive days after hospitalization	Deferoxamine mesylate might accelerate hematoma absorption and inhibit edema
Selim M et al., 2019 [14]	144	Hemorrhagic stroke	59.00 ± 3.33	61.1%	32 mg/kg/d, maximum daily dose: 6000 mg/d	Intravenous infusion	3 consecutive days after hospitalization	DFO would be futile to significantly improve the chance of good outcome at day 90

### Meta-analysis

To obtain a more accurate outcome of meta-analysis, we tested the statistical difference of three continuous variables before administration of DFO by ANOVA respectively (Table 2). There was statistical difference in NIHSS scores before administration of DFO (*F* = 4.112, *p* < 0.001). Thus, we calculated the reduction of NIHSS scores before administration of DFO and at 30 days after onset (Table 3). However, HV before administration of DFO (*F* = 1.964, *p* = 0.086) and RPHE before administration of DFO (*F* = 2.134, *p* = 0.064) had no statistical difference. Their data at 7 days after onset were directly used in meta-analysis.

**Table 2:** NHISS scores at 30 days after onset, HV at 7 days after onset, and RPHE at 7 days after onset between DFO and control groups

Studies	Size	DFO group		Control group		*F*	*P*
		Before	After	Before	After		
NIHSS scores (mean ± SD)							
Selim M 2010 [12]	7	14.4 ± 3.72	8.83 ± 4.96	13.00 ± 2.82	9.25 ± 8.48	4.112	0.001
Selim M et al., 2011 [11]	20	13.25 ± 8.75	3.75 ± 2.75	11.75 ± 5.25	4.25 ± 2.75		
Yu Y et al., 2015 [13]	21	9.10 ± 4.60	3.80 ± 3.90	8.70 ± 5.40	4.20 ± 4.10		
Selim M et al., 2019 [14]	144	13.00 ± 4.50	3.00 ± 2.83	13.00 ± 4.30	4.00 ± 3.81		
HV (mean ± SD, ml)							
Selim M et al., 2011 [11]	20	17.415 ± 11.250	9.155 ± 4.245	17.275 ± 7.790	11.650 ± 7.245	1.964	0.086
Yu Y et al., 2015 [13]	21	15.200 ± 8.800	8.220 ± 6.850	12.600 ± 10.300	7.780 ± 7.750		
Yu J et al., 2017 [10]	47	12.770 ± 6.360	5.220 ± 3.330	12.570 ± 7.790	8.380 ± 4.110		
RPHE (mean ± SD)							
Selim M et al., 2011 [11]	20	0.27 ± 0.21	0.66 ± 0.45	0.35 ± 0.21	1.98 ± 0.68	2.134	0.064
Yu Y et al., 2015 [13]	21	0.31 ± 0.23	0.68 ± 0.50	0.28 ± 0.29	1.96 ± 1.03		
Yu J et al., 2017 [10]	47	0.19 ± 0.15	0.42 ± 0.42	0.27 ± 0.20	1.88 ± 0.95		

*NIHSS*, National Institute of Health Stroke Scale. *HV*, hematoma volume. RPHE, relative perihematomal edema. *DFO*, desferrioxamine mesylate. *SD*, standard deviation. *Before*, before administration of DFO. *After*, HV and RPHE at 7 days after onset of ICH, NIHSS scores at 30 days after onset of ICH. *F*, detection of data before administration of DFO via analysis of variance. *p*, *p*-value

**Table 3:** Reduction of NIHSS scores (mean ± SD)

Studies	Size	DFO group	Control group
Selim M 2010 [12]	7	5.31 ± 4.47	3.75 ± 7.48
Selim M et al., 2011 [11]	20	9.50 ± 7.75	7.50 ± 4.55
Yu Y et al., 2015 [13]	21	5.30 ± 4.29	4.50 ± 4.88
Selim M et al., 2019 [14]	144	10.00 ± 3.94	9.00 ± 4.08

*NIHSS*, National Institute of Health Stroke Scale. *SD*, standard deviation. *DFO*, desferrioxamine mesylate

**Fig. 3:**
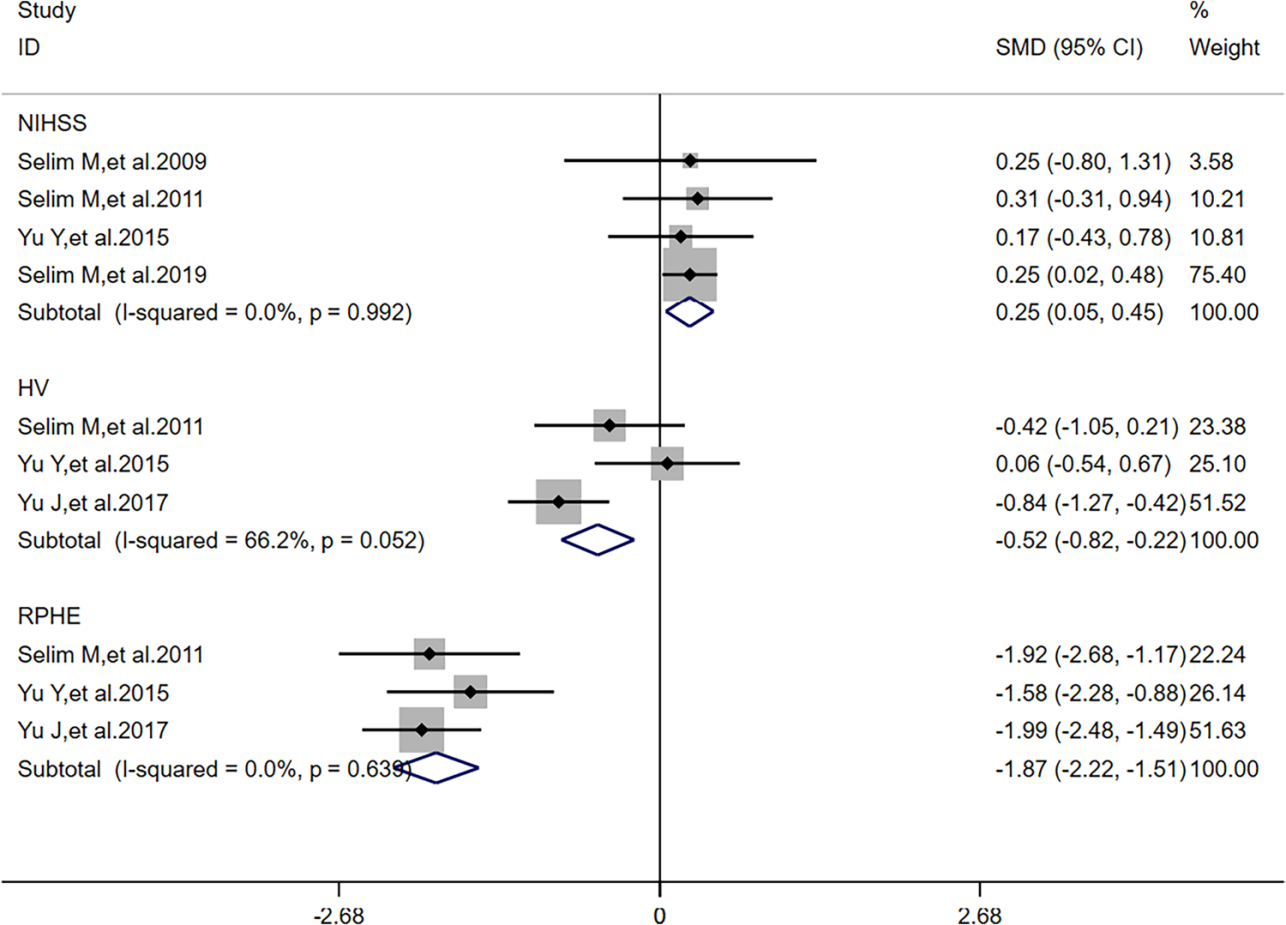
Forest plots for the comparisons of NHISS scores/HV/RPHE between DFO and control groups. NIHSS: National Institute of Health Stroke Scale. HV: hematoma volume. RPHE: relative perihematomal edema. DFO: desferrioxamine mesylate. SMD: standard mean difference. 95%*CI*: 95% confidence interval. *p*: *p*-value

There was no heterogeneity (*I*^2^ = 0) in meta-analysis of NIHSS scores and RPHE (Fig. 3). There was significant difference of RPHE (*SMD* =  − 1.87, 95%*CI* =  − 2.22 ~  − 1.51, *p* = 0.992) and reduction of NIHSS scores (*SMD* = 0.25, 95%*CI* = 0.05 ~ 0.45, *p* = 0.639) between DFO and control groups. Obvious heterogeneity (*I*^2^ = 66.2%) was found in meta-analysis of HV. There was no significant difference of HV (*SMD* =  − 0.44, 95%*CI* =  − 0.98 ~ 0.11, *p* = 0.052) between DFO and control groups.

### Publication bias and sensitivity analysis

**Fig. 4:**
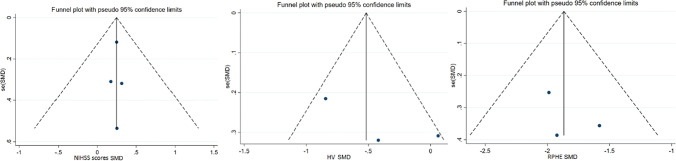
Funnel plots of risk of publication bias of NHISS scores/HV/RPHE. NIHSS: National Institute of Health Stroke Scale. HV: hematoma volume. RPHE: relative perihematomal edema. SMD: standard mean difference

There was no publication bias according to symmetrical distribution in funnel plots of HV at 7 days after onset, RPHE at 7 days after onset, and reduction of NIHSS scores at baseline and 30 days after onset (Fig. 4). There was no significant difference of reduction of NIHSS (*I*^2^ = 0, *SMD* = 0.24, 95%*CI* =  − 0.16 ~ 0.65, *p* = 0.951) between DFO and control groups after deleting the study with the highest quality [14]. There was significant difference of HV (*I*^2^ = 17.5%, *SMD* =  − 0.70, 95%CI =  − 1.09 ~  − 0.30, *p* = 0.271) between DFO and control groups after the study with the highest quality was omitted [13]. There was significant difference of RPHE (*I*^2^ = 0, *SMD* =  − 1.97, 95%*CI* =  − 2.38 ~  − 1.55, *p* = 0.885) between DFO and control groups after the study with the highest quality was omitted [13].

### Influence analysis and subgroup analysis

**Fig. 5:**
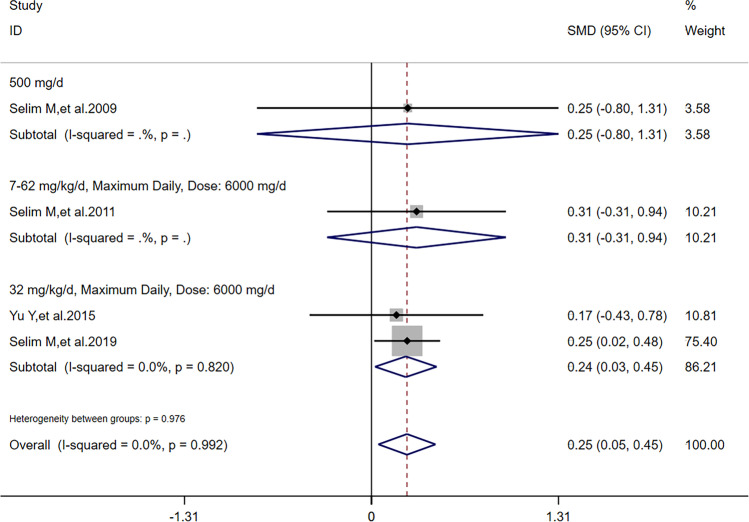
Subgroup analysis of NHISS scores referring to different dose of DFO. NIHSS: National Institute of Health Stroke Scale. DFO: desferrioxamine mesylate. SMD: standard mean difference. 95%*CI*: 95% confidence interval. *p*: *p*-value

**Fig. 6:**
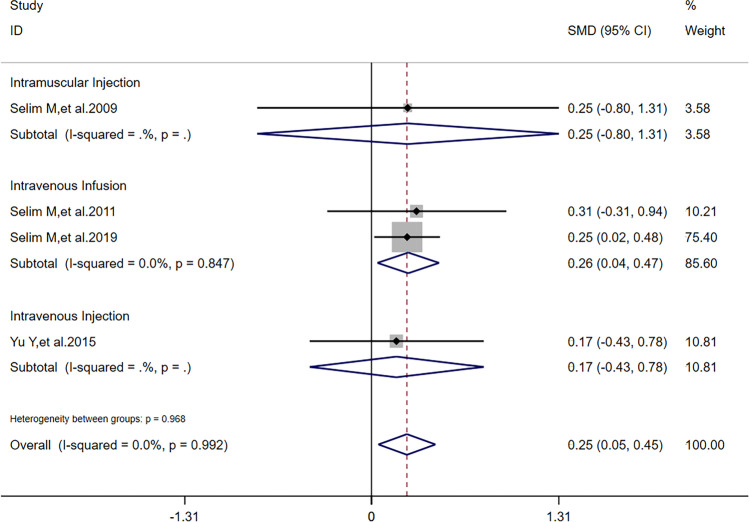
Subgroup analysis of NHISS scores referring to different routes of administration of DFO. NIHSS: National Institute of Health Stroke Scale. DFO: desferrioxamine mesylate. SMD: standard mean difference. 95%*CI*: 95% confidence interval. *p*: *p*-value

**Fig. 7:**
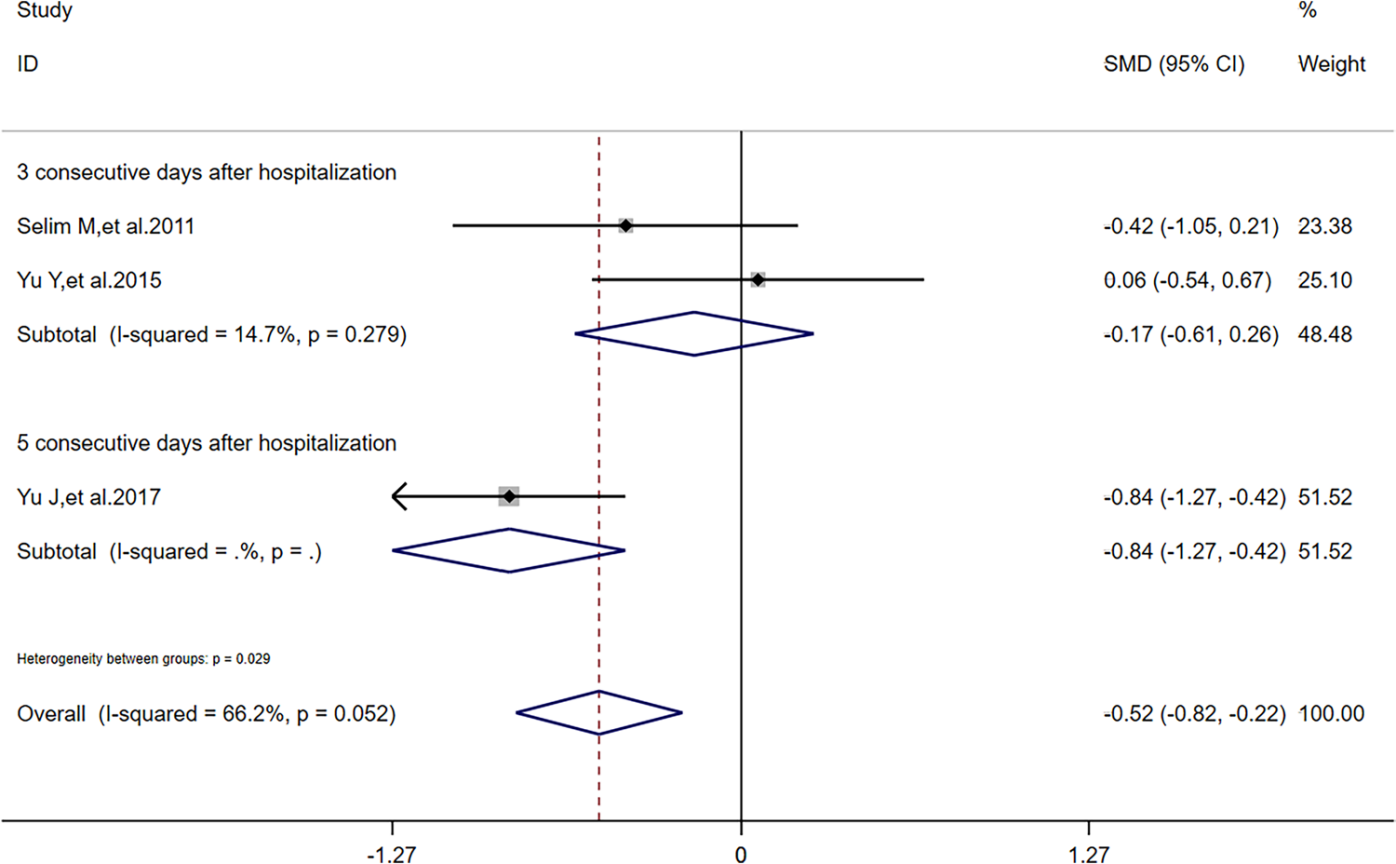
Subgroup analysis of HV referring to different time of treatment. HV: hematoma volume. SMD: standard mean difference. 95%*CI*: 95% confidence interval. *p*: *p*-value

**Fig. 8:**
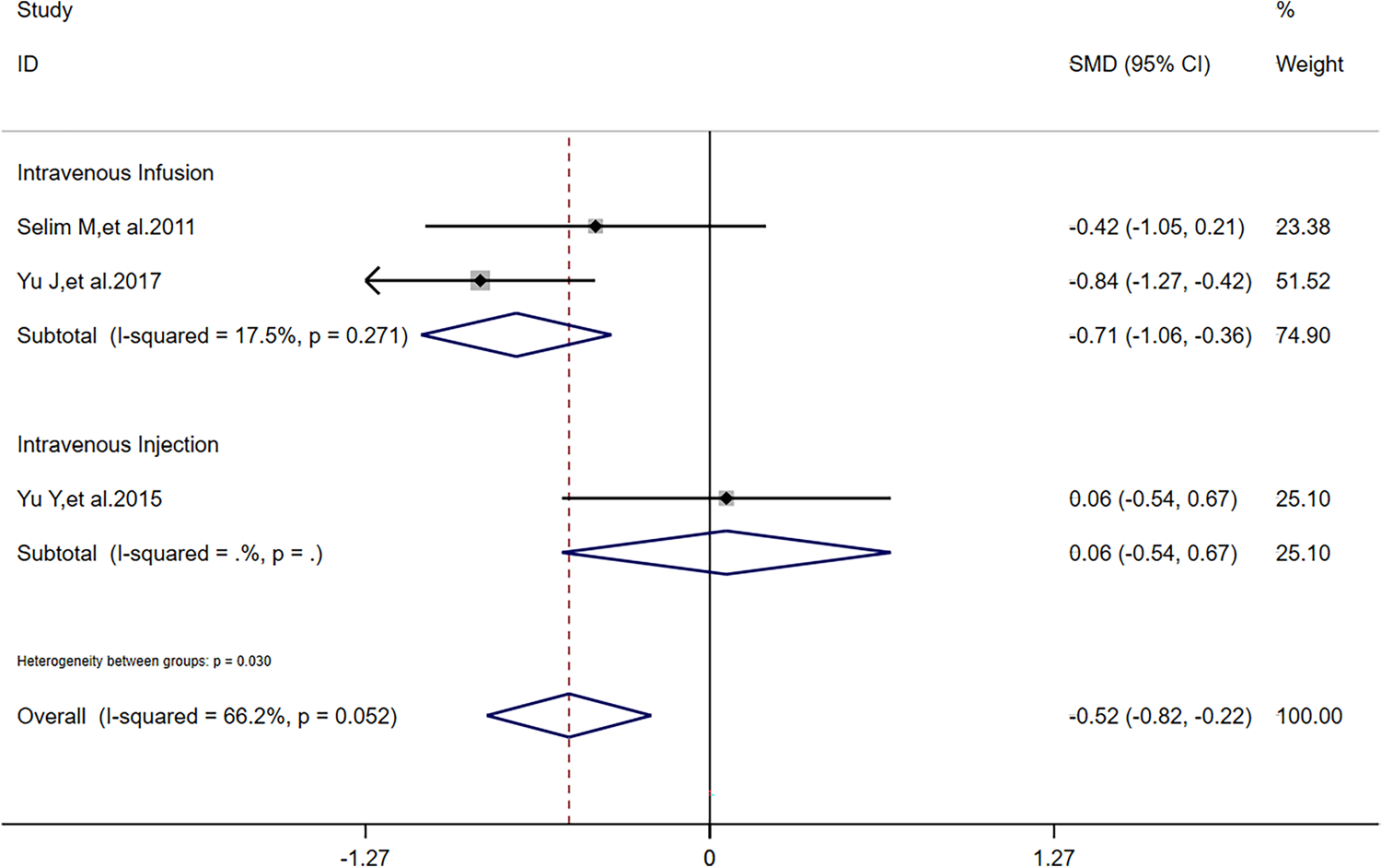
Subgroup analysis of HV referring to different routes of administration of DFO. HV: hematoma volume. DFO: desferrioxamine mesylate. SMD: standard mean difference. 95%*CI*: 95% confidence interval. *p*: *p*-value

**Fig. 9:**
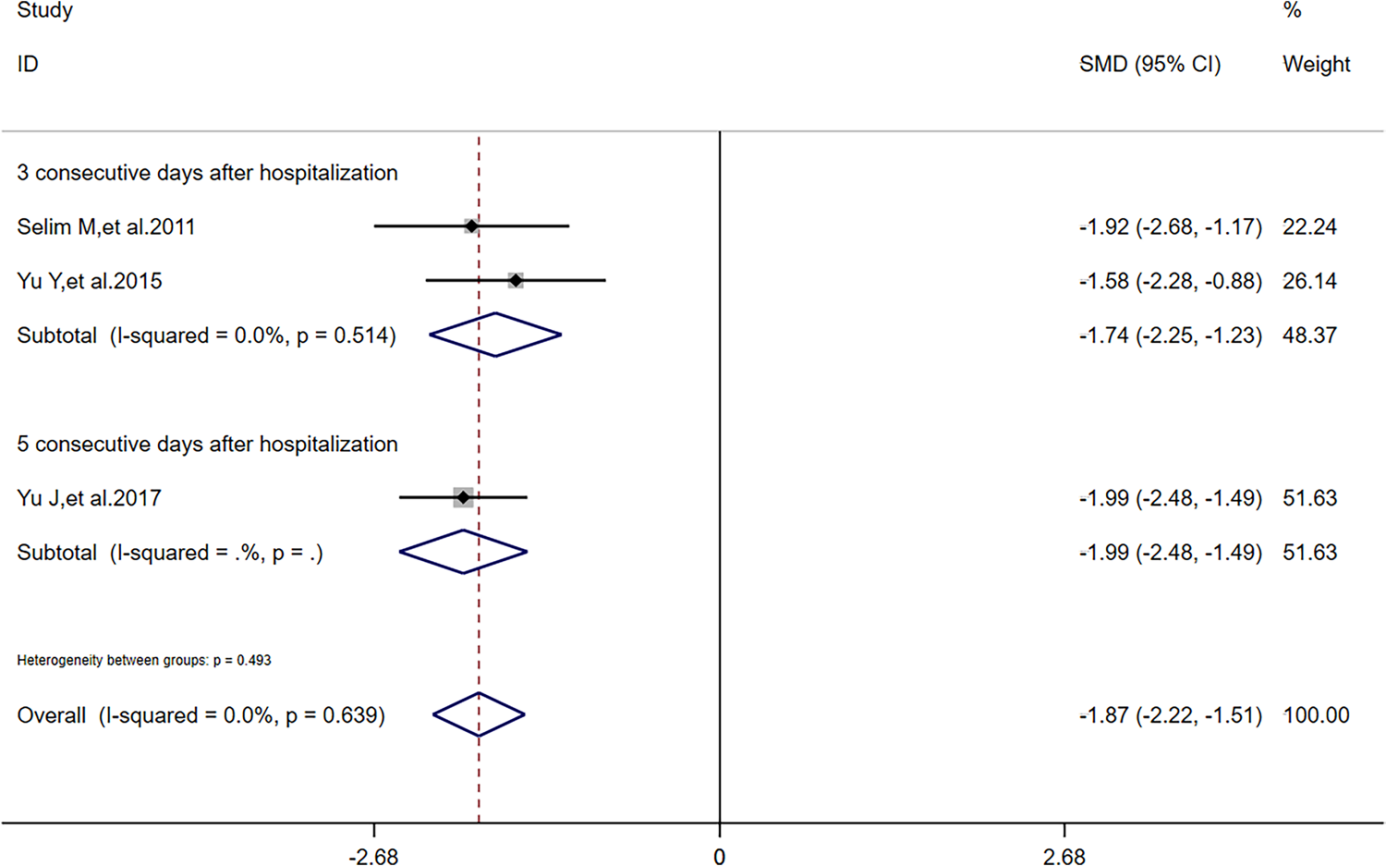
Subgroup analysis of RPHE referring to different time of treatment. RPHE: relative perihematomal edema. SMD: standard mean difference. 95%*CI*: 95% confidence interval. *p*: *p*-value

**Fig. 10:**
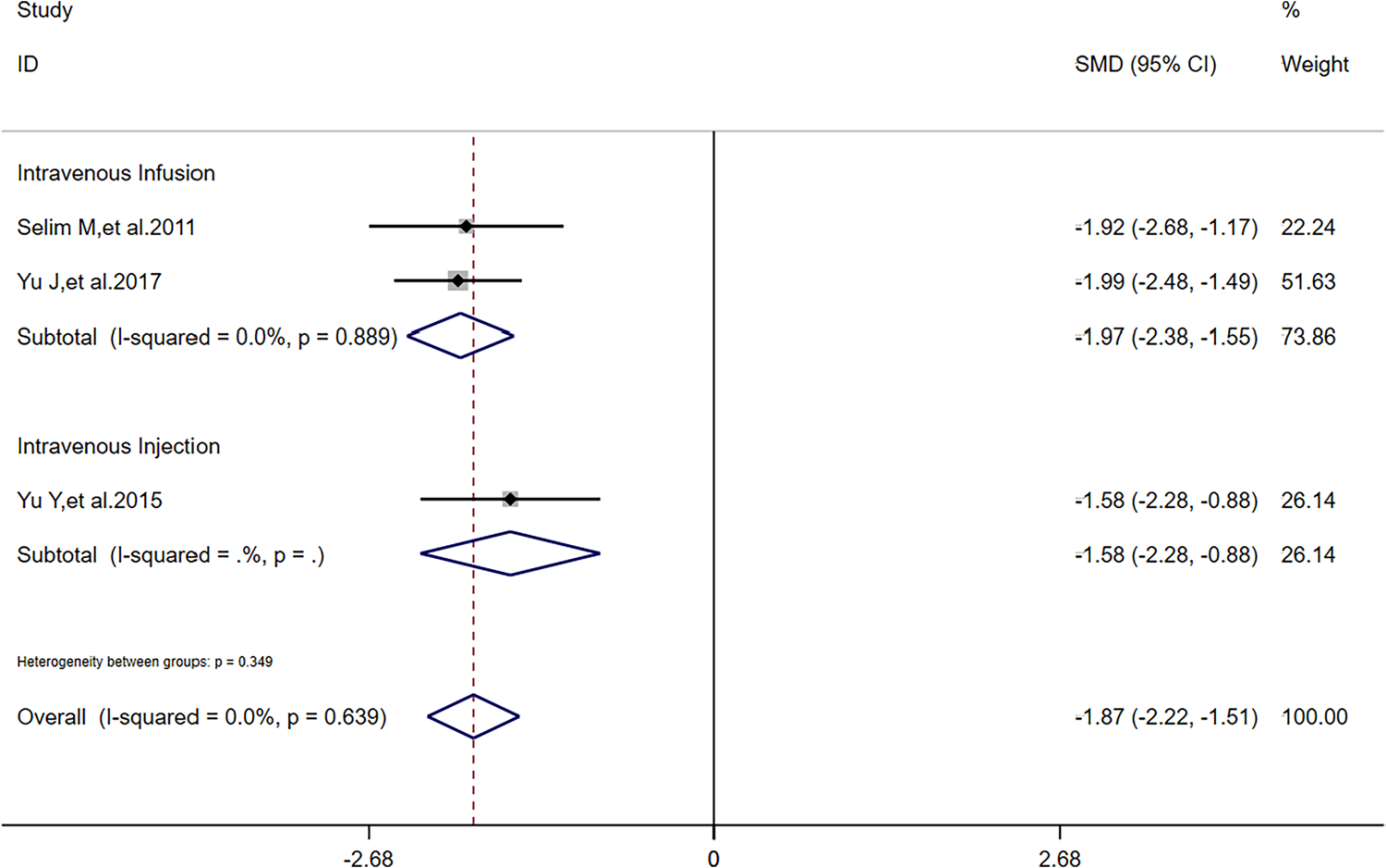
Subgroup analysis of RPHE referring to different routes of administration of DFO. RPHE: relative perihematomal edema. DFO: desferrioxamine mesylate. SMD: standard mean difference. 95%*CI*: 95% confidence interval. *p*: *p*-value

After sensitivity analysis, solely referring to studies’ quality, we found that reduction of NIHSS scores had a difference outcome without heterogeneity; HV had the same outcome and its heterogeneity was also lower than before; RPHE had the same outcome without heterogeneity. To detect the nature of this phenomenon, in addition to one study [11] in all the 5 articles reported that dose of DFO was correlated to HV, RPHE, and NHISS scores, we performed subgroup analysis according to critical factors in using DFO: dose (including maximum daily dose), route of administration, and time of treatment. Referring to characters of studies (Table 1) and extraction of data (Tables 2 and 3), we performed subgroup analysis of HV, RPHE, and reduction of NIHSS scores, respectively. If dose was 32 mg/kg/d (maximum daily dose: 6000 mg) and time of treatment was 3 consecutive days after hospitalization, there was significant difference of reduction of NIHSS scores (*I*^2^ = 0, *SMD* = 0.24, 95%*CI* = 0.03 ~ 0.45, *p* = 0.820) between DFO and control groups (Fig. 5). If route of administration was intravenous infusion and time of treatment was 3 consecutive days after hospitalization, there was significant difference of reduction of NIHSS scores (*I*^2^ = 0, *SMD* = 0.26, 95%*CI* = 0.04 ~ 0.47, *p* = 0.846) between DFO and control groups (Fig. 6). If time of treatment was 3 consecutive days after hospitalization, there was no significant difference of HV (*I*^2^ = 14.7%, *SMD* =  − 0.17, 95%*CI* =  − 0.61 ~ 0.26, *p* = 0.279) between DFO and control groups (Fig. 7). If route of administration was intravenous infusion, there was significant difference of HV (*I*^2^ = 17.5%, *SMD* =  − 0.70, 95%*CI* =  − 1.09 ~  − 0.30, *p* = 0.271) between DFO and control groups (Fig. 8). If time of treatment was 3 consecutive days after hospitalization, there was no significant difference of RPHE (*I*^2^ = 0, *SMD* =  − 1.74, 95%*CI* =  − 2.25 ~  − 1.23, *p* = 0.514) between DFO and control groups (Fig. 9). If route of administration was intravenous infusion, there was significant difference of RPHE (*I*^2^ = 0, *SMD* =  − 1.97, 95%*CI* =  − 2.38 ~  − 1.55, *p* = 0.885) between DFO and control groups (Fig. 10).

## Discussion

Numerous animal studies have confirmed that DFO could be used to treat ICH [15–18]. Formation of PHE is mainly due to inflammation and broken blood–brain barrier around hematoma lesion [19]. Hematoma absorption in a short period of time might be the key to improve the prognosis of ICH patients. Iron is a critical component of hematoma tissue. DFO might promote hematoma absorption and reduction of PHE via accelerating iron metabolism. Some randomized controlled trials have concluded that infusions of DFO after ICH were feasible, safe, and well-tolerated (Table 1). In other words, DFO might reduce edema and improve neurological function by controlling hematoma. Only the study [11] in all the 5 articles reported that patients whose dose was 32 mg/kg had the lowest NHISS scores at 30 days after onset of ICH and the most obvious decrease of HV at 7 days after onset of ICH; patients whose dose was 57 mg/kg had the slowest development of RPHE at 7 days after onset of ICH. In addition, time of treatment and routes of administration could influence the real-time change of blood concentration of DFO, which might influence the efficacy of treatment. Therefore, we not only performed sensibility analysis referring to studies’ quality but also performed the influence analysis and subgroup analysis referring to critical factors in using DFO.

We found that DFO could improve hematoma absorption in ICH patients affirmatively with an unstable outcome of sensibility analysis. It interested us that after the study with the highest quality was omitted, meta-analysis of HV had the lower heterogeneity. In one influence analysis of HV, we found that DFO did not effectively decrease HV in ICH patients whose time of treatment was 3 consecutive days after hospitalization. Interestingly, this result was acquired under the premise that the study [10] with the lowest quality had been omitted. Moreover, we considered that the nature of selection bias of the study [10] was not only patients were mostly male, but also patients’ causes of ICH included craniocerebral injury, which meant these ICH patients also had focal cerebral contusion. Although focal cerebral contusion was also brain space–occupying lesion caused by hematoma, it damaged brain tissue directly, which might promote DFO to be diffused in lesion. The data extracted from this article showed that decrease of HV and RPHE was the most obvious in all the 5 articles included, while the dose of DFO was lower, which might prove that diffusion of DFO is hundred-percent to benefit the efficacy of treatment. Therefore, with regard to decrease HV, time of treatment of 3 consecutive days might be not enough in ICH patients who suffered hemorrhagic stroke. Time of treatment might be an influence factor of HV decreased by DFO. In another influence analysis of HV, we found that DFO effectively decreased HV in ICH patients whose routes of administration were intravenous infusion. Amazingly, this result was acquired under the premise that the study [13] with the highest quality had been omitted. Apart to study’s quality, comparing to intravenous injection, we considered that intravenous infusion could make blood concentration of DFO stable, which benefited chelating iron via DFO completely. Therefore, intravenous infusion might be the better route of administration of DFO in ICH patients. In sum, DFO might decrease HV at 7 days after onset of ICH effectively; in addition, appropriate longer time of treatment and intravenous infusion might both benefit this effect.

In meta-analysis of NIHSS scores, we found that DFO could improve neurological foundation of ICH patients at 30 days after onset. Although heterogeneity was still zero, the result of sensibility analysis was disparate to it. To detect the nature of contradiction, we performed influence analysis. We found that under the premise of time of treatment of 3 consecutive days after hospitalization, DFO effectively decreased NIHSS scores in ICH patients whose dose was 32 g/kg/d (maximum daily dose: 6000 mg); DFO effectively decreased NIHSS scores in ICH patients whose routes of administration were intravenous infusion. Moreover, both of this two subgroup analysis excluded the article with the lowest quality [12]. Therefore, we considered that DFO administrated via intravenous infusion with the dose of 32 mg/kg/d (maximum daily dose: 6000 mg; time of treatment: 3 consecutive days after hospitalization) might effectively decrease NIHSS scores at 30 days after onset of ICH. Dose and route of administration were influence factors of efficacy of DFO. Although RPHE was not an absolute variable, it could reflect the development of PHE depending on the changes of hematoma lesion. According to the meta-analysis of RPHE, we concluded that development of PHE in DFO group was slower than that in control group at 7 days after onset definitely. Only results of RPHE had a stable outcome of sensibility analysis. In addition, causes of ICH, dose, time of treatment, and routes of administration did not influence the efficacy of DFO in treatment of ICH patients. Moreover, there might be other pathways to achieve its treatment effect. Recent preclinical studies have reported that DFO could improve brain dysfunction, rescue dendritic axon damage, and inhibit microglia activation and attenuation of blood–brain barrier [20–22]. Theoretically speaking, DFO’s molecular weight was lower than the maximum of molecular weight which could go through blood–brain barrier. Iron from hematoma could promote the development of PHE via another route: cortical superficial siderosis, which meant the iron deposited in brain tissue [23]. Due to peroxidation of iron in brain tissue, PHE could influence patients’ long-term prognosis including neurological foundation. According to our study, DFO decreased PHE and NIHSS scores, which might indirectly show that DFO enter brain tissue via blood–brain barrier and perform its therapeutic effect and both of dose and route of administration were influence factors of efficacy of DFO. Only one study [11] reported the correlation of adverse reaction or severe adverse events and dose of DFO; other 4 articles directly showed no any adverse reaction happened on patients or did not mentioned this. The study [11] showed that only patients whose dose was 32 mg/kg had no any adverse reaction of DFO. Therefore, we did not perform the meta-analysis about safety of using DFO. In sum, referring to characters of studies included, 20–32 mg/kg/d dose of DFO (maximum daily dose ≤ 6000 mg, time of treatment ≤ 5 consecutive days after hospitalization) might be safe.

Only five randomized controlled trials were included in the meta-analysis finally. Furthermore, three of them had the same first author. One study [10] was assessed to have high risk in item of selection bias, which might affect the results of the analysis when the number of articles was small. In addition, data of NIHSS scores used in this meta-analysis were processed via mathematical transformation and the quantization of PHE was defined to a relative number. The outcome might be affected due to non-original or non-absolute data. In addition, time points of data in the original articles were in the early period of treatment in ICH patients, such as HV measured at 7 days after onset and NIHSS scores assessed at 30 days after onset, which would affect the conclusion of our meta-analysis. The article [6] only included animal models of ICH to perform the systematic review and meta-analysis [6]. They found that DFO reduced the brain water content in animal models of ICH and improved the neurobehavioral score; DFO was most efficacious when administered 2–4 h after ICH at a dose of 10–50 mg/kg depending on species, and this beneficial effect remained for up to 24 h postinjury. Though total conclusion was the same as our study, there was difference between preclinical research and clinical research. We found that the method with the best efficacy and safety of DFO in treatment of ICH might be dose: 32 mg/kg/d, maximum daily dose: 6000 mg/d, period of treatment: 3 consecutive days after hospitalization, and route of administration: intravenous infusion; under this solution, HV and PHE at 7 days after onset of ICH and NIHSS scores at 30 days after onset of ICH were decreased. Only two studies were included in the article (Zeng L et al. 2018) [24]. It only included hemorrhagic stroke patients as ICH patients and reported that DFO was an effective treatment for edema in patients with ICH, which was in accordance of our conclusion. But the article did not perform meta-analysis as the data evidence to support systemic review. Because focal cerebral contusion was also brain space–occupying lesion caused by hematoma, in addition to that, our study included craniocerebral injury patients. The article concluded that elevated ferritin levels were associated with higher mortality of hemorrhagic stroke, especially in Corona Virus Disease 2019 patients as a complication [25]. Mostly, DFO decreased ferritin via chelating its iron, which indirectly confirmed the conclusion from our study that DFO might be used in the treatment of ICH. In future, we hoped that levels of iron in urine or feces and ferritin should be detected as the indicators of metabolic used in clinical research about DFO treating ICH.

After duplication was excluded, 23 conference abstracts had been excluded. The exclusion of conference abstracts, where small trials can be reported without a formal publication, might also cause bias. All the 23 conference abstracts were reviews, comments, protocols, or abstracts which did not conform to our theme, which is shown on Fig. 1. We hope that more small trials of DFO treating ICH might be encouraged in academic conferences. Among all the articles included finally, 4 articles reported outcomes of modified Rankin Scale (mRS), 2 articles reported outcomes of modified Barthel Index (mBI), and only 1 article reported the outcome of Glasgow Outcome Scale (GOS). Except to GOS, we should calculate overall standard mean difference with its 95% confidence interval to perform meta-analysis of mRS or mBI. But no article reported the mBI at baseline. Only 1 article reported the mRS at baseline. Finally, we could not perform the meta-analysis of clinical outcome. Fortunately, 4 articles reported outcomes of NIHSS including them at baseline. And we considered that change of neurological function could indirectly reflect clinical outcome. The lack of clinical outcome might be the fact as a limitation of our systematic review value. We advocated that outcomes of clinical researches should be reported completely, especially in randomized controlled trials.

## Conclusion

Apart of the main limit that few of studies were included in our study, DFO (32 mg/kg/d, maximum daily dose: 6000 mg, time of treatment: 3 days after hospitalization) reduced HV and PHE in ICH patients at 7 days after onset and improve neurological function at 30 days after onset efficiently and safely. DFO might be a new route of improving treatment of ICH.
